# Sensor Modeling for Underwater Localization Using a Particle Filter

**DOI:** 10.3390/s21041549

**Published:** 2021-02-23

**Authors:** Humberto Martínez-Barberá, Pablo Bernal-Polo, David Herrero-Pérez

**Affiliations:** 1 Facultad de Informática, University of Murcia, 30100 Murcia, Spain; humberto@um.es (H.M.-B.); pablo.bernal.polo@gmail.com (P.B.-P.); 2 Technical University of Cartagena, Campus Muralla del Mar, 30202 Cartagena, Murcia, Spain

**Keywords:** underwater vehicle frameworks, underwater localization, uncertainty modeling, multi-sensor fusion, navigation, sonar

## Abstract

This paper presents a framework for processing, modeling, and fusing underwater sensor signals to provide a reliable perception for underwater localization in structured environments. Submerged sensory information is often affected by diverse sources of uncertainty that can deteriorate the positioning and tracking. By adopting uncertain modeling and multi-sensor fusion techniques, the framework can maintain a coherent representation of the environment, filtering outliers, inconsistencies in sequential observations, and useless information for positioning purposes. We evaluate the framework using cameras and range sensors for modeling uncertain features that represent the environment around the vehicle. We locate the underwater vehicle using a Sequential Monte Carlo (SMC) method initialized from the GPS location obtained on the surface. The experimental results show that the framework provides a reliable environment representation during the underwater navigation to the localization system in real-world scenarios. Besides, they evaluate the improvement of localization compared to the position estimation using reliable dead-reckoning systems.

## 1. Introduction

Nowadays, underwater vehicles allow us access to restricted areas and traditionally dangerous environments for human divers, such as deep seabed and under the ice. These vehicles can perform many tasks in a broad spectrum of applications, such as inspection, repair, and maintenance [1] in defense, oil and gas, and cable surveying, to name but a few. Underwater vehicles incorporating a certain degree of autonomy usually rely on proprioceptive sensors [2], such as an inertial navigation system (INS) integrated with Doppler velocity logs (DVLs) [3]. This is because the submerged vehicle cannot detect the electromagnetic signals provided by the global navigation satellite system (GNSS). However, these proprioceptive sensors suffer from drift and biases, leading to growing position uncertainty as the vehicle navigates. This fact makes unfeasible underwater dead-reckoning navigation. For this reason, several works in the literature combine the information of proprioceptive sensors to external positioning systems [4].

Acoustic positioning systems are the most used underwater external positioning approaches, from long baseline (LBL) [5,6,7] to sort baseline (SBL) [8] and ultrashort baseline (USBL) [9,10]. However, these sensors suffer from multipath Doppler effects and thermoclines, which induce acoustic reflection effects. These underwater localization systems also require the deployment of a network of sea-floor mounted baseline transponders, often in the perimeter of the workplace area, for LBL or a support vessel with the transponders following the vehicle for SBL and USBL. We can also use optical or sonar sensors to identify specific landmarks in the environment and use them to locate the underwater vehicle using an *a priori* representation of the environment [11]. A significant advantage of this approach is the cheap cost with a minimum modification of the workplace. The effectiveness of localization based on an a priori known representation of the environment depends on the wealth of useful information. We should bear in mind that optical and sonar sensors provide a short range of high-resolution uncertain sensing readings, mainly due to the different factors affecting their uncertainty, such as multipath reflections and poor underwater visibility. Therefore, the modeling, processing, and fusing of underwater sensor readings are of paramount importance to build a reliable representation of the submarine environment. The reliability of such a perception is a key for tracking and location purposes.

Tracking and localization methods aim to fuse uncertain position measurements to estimate the variable of interest with different assumptions about the representation of the vehicle’s location. The techniques based on variants of the Kalman filter and Sequential Monte Carlo (SMC) method are the most popular tracking and localization methods, respectively. The Kalman filter is a recursive state estimator of a discrete-time controlled process governed by a linear stochastic differential equation. It is the minimum variance state estimator for linear dynamic systems with Gaussian noise and the minimum variance linear state estimator for linear dynamic systems with non-Gaussian noise. We usually use these methods for tracking the vehicle position in underwater scenarios. We can mention the Extended Kalman filter (EKF) [12,13,14] and the unscented Kalman filter (UKF) [9,15]. However, we have to remark that these techniques are suboptimal state estimators for a non-linear system. One of the main advantages of such tracking techniques is that they represent the state and its uncertainty using a Gaussian distribution. This representation facilitates the efficient implementation of the filter with a reduced computational cost. However, they are not able to recover from divergences in the recursive estimation process. The localization approaches based on the SMC method [16,17,18] are robust to uncertain and incoherent information, allowing recovery from divergences in the state estimation process. Nevertheless, they suffer from severe computational requirements. This fact is particularly for large and complex domains, where we require numerous samples to represent complex stochastic distributions of the state-space model.

In this paper, we present a framework for processing, modeling, and fusing underwater sensor signals to obtain a reliable representation of the submarine environment around the vehicle. We use such perceptions for localization purposes during underwater navigation. We process the raw sensor readings to detect the features surrounding the reference vehicle. This processing allows us to filter measures that do not match with features. We also incorporate uncertainty representation to the detected features. We use this information to fuse feature perceptions between them, which allows us to remove redundant information and maintain a coherent perception in consecutive observations. The underline idea is to consider the uncertainty of the perception to propagate it to the localization method.

In particular, we present the feature extraction from buffered data of underwater observations using optical and sonar sensors. We use a mechanism to verify these data by coherent consecutive and redundant perceptions. We update the uncertainty of such buffered perceptions induced by the movement of the vehicle and aging. We also remove the observations from the buffer by aging and disparity. We propagate the uncertainty of the sensor readings to the set of features surrounding the underwater vehicle. This set of features provides a coherent local representation of the environment. We also update and remove these features using the factors previously mentioned. The use of this local buffer representation allows us to filter out inconsistent exteroceptive sensory information. The reliability of this local representation is of paramount importance because incoherent perceptions can deteriorate the position estimations. We use the extracted features from noisy underwater sensors to feed the update stage of a particle filter localization method. We have to remark that we can use the presented sensor modeling techniques with other localization approaches.

We organize the manuscript as follows. Section 2 describes the underwater platform used in this work. It details the sensory system incorporated into the vehicle and the description of the hardware architecture. We devote Section 3 to the processing and modeling of underwater sensor signals to provide a reliable representation during submarine navigation. This representation includes information about the uncertainty, which is updated using the factors that induce noise and imprecisions to such environment representation. We propagate such uncertain information to a recursive state estimator. This fact allows us to estimate both the location of the submerged vehicle and its uncertainty. Section 4 presents the modified sequential Monte Carlo (SMC) method as a recursive estimator to obtain the variable of interest. Section 5 shows the experimental results evaluating the proposed framework. We assess the processing, modeling, and sensor fusion of underwater sensor signals using the localization method. Finally, Section 6 presents the conclusion and the future works of the proposal.

## 2. Underwater Platform

**Figure 1:**
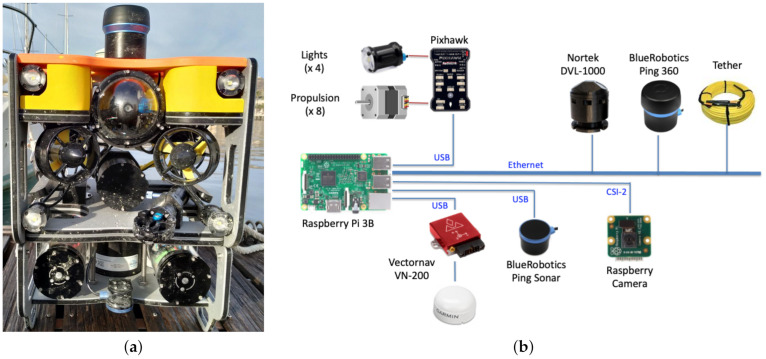
(**a**) Modified Sibiu Pro underwater vehicle from Nido Robotics company, and (**b**) hardware architecture and sensory system.

We use the commercial platform Sibiu Pro underwater vehicle from Nido Robotics company incorporating new sensors and electronics for testing the developments presented in this work. The Sibiu Pro standard platform is a fully operational underwater vehicle operated with an umbilical cable. It is specially designed for the inspection and maintenance of submerged systems. The propulsion system uses a Thrust Vector Control (TVC) with three propellers that allow the vehicle to move/rotate in any direction combining them. It also incorporates a 1080p camera with 1500 lumens lights to obtain a clear image in low-light environments. Figure 1a shows the Sibiu Pro platform incorporating the sensory system used in this work; the sonar scanner, the Doppler Velocity Logger (DVL), and the GPS.

Figure 1b shows the hardware architecture and the sensory system incorporated into the platform to increase the functionalities. As proprioceptive sensors, we include a VectorNav VN-200 inertial navigation system and a Nortek DVL-1000. The former combines MEMS inertial sensors and a high-sensitivity GNSS receiver to estimate position, velocity, and orientation. It also allows us to obtain the GPS location on the surface in UTM coordinates. The latter is an acoustic instrument that can estimate the velocity relative to the bottom or to the surface. The combination of both systems provides accurate dead-reckoning estimations obviating the GNSS receivers. As exteroceptive sensors, we include a Blue Robotics Ping sonar (Ping 360) and an 8-megapixels Sony IMX219 Raspberry camera. The former is a mechanical scanning sonar providing underwater acoustic imaging with 50 m range, 300 m depth rating, and an open-source software interface using Ethernet. The latter replaces the camera of the Sibiu Pro platform with higher specifications. We install the software that communicates with propulsion, lights, and sensors in a Raspberry Pi 3B, it using the interfaces indicated in Figure 1b. We currently perform the intensive computation in an external CPU that communicates with the Raspberry Pi using TCP/UDP protocols through the Ethernet of the umbilical cable.

## 3. Sensor Modeling

We present the processing and modeling of different underwater sensor signals to provide a reliable representation of the environment for underwater localization in structured environments. The information provided by these underwater sensors is often affected by diverse sources of uncertainty that can deteriorate the positioning and tracking. In particular, we present the image processing of artificial markers using an 8-megapixels Sony IMX219 Raspberry camera and the processing of the data provided by the mechanical sonar scanner Ping360 of Blue Robotics. The processing of the feature detection techniques aims to filter out noisy information and inconsistencies in sequential observations.

### 3.1. Visual Perception of Landmarks

We use a fiducial marker system specially appropriated for localization in structured environments. In particular, we distribute Aruco markers [19,20] along the structured environment to take references during the navigation. The perception of such landmarks allows us to improve the localization accuracy operating in the underwater environment. Following [19], we adopt the process for marker detection from grayscale images consisting of image segmentation, contour extracting and filtering, marker code extraction, and marker identification. The image segmentation consists of the extraction of the most prominent contours in the grayscale image. We use a local adaptive thresholding strategy based on the analysis of neighboring pixels for their robustness to different lighting conditions. The contour extraction stage detects polygons with four-vertices. We also discard four-vertex polygons contained in other quadrilateral features leaving only the external ones. Then, the marker code extraction stage removes the perspective projection computing the homography matrix. We then tessellate the resulting pixels assigning zero or one value to each cell of a regular 11 × 11 grid. Finally, the marker identification stage matches the tessellated image with the dictionary of markers generated for the structured environment. We require four different identifiers for each Aruco marker generated (one for each possible rotation).

**Figure 2:**
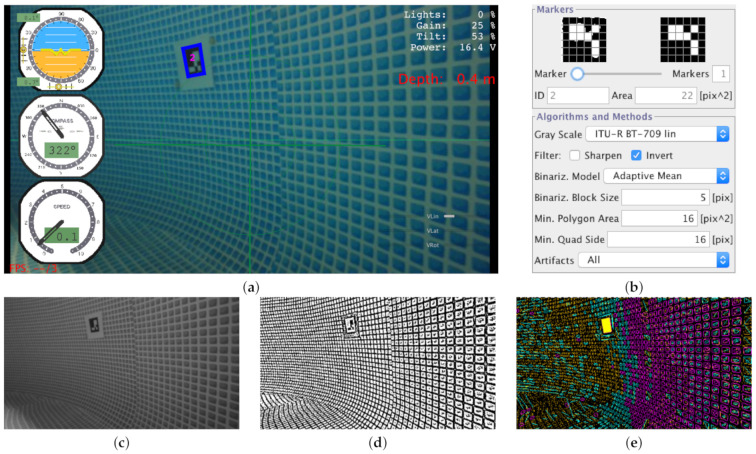
(**a**) Aruco marker with ID 2 detected by the on-board camera and (**b**) the on-line parameter configuration of maker recognition process with the (**c**) grayscale image, (**d**) binarized image, and (**e**) polygon extraction stage with marker identification.

Although the concept of marker detection is simple, there are several parameters to control the detection process. Besides, these parameters are strongly dependent on the image resolution. For these reasons, we have developed configuration tools to perform the calibration and configuration while the vehicle is operating underwater. Figure 2 shows an example of the vision processing for detecting the Aruco markers and the calibration tools used to facilitate the configuration. Figure 2a shows an image captured by the on-board camera of the underwater vehicle operating in a swimming pool with landmarks distributed throughout its walls. These landmarks consist of 11 × 11 Aruco markers printed at 12 × 12 cm with a plastic film covering to make them waterproof. The image resolution is 480 × 360, which allows us to process 15 frames per second from the computer operating the vehicle. Figure 2b shows the interface for the on-line configuration of the parameters of the processing. This tool allows us to modify the configuration of Aruco markers with wrong identification as the vehicle operates. The proper tunning of marker detection increases the robustness of the perception, which is of paramount importance because a lack of it could degrade the localization process.

Figure 2c shows the grayscale image used for detecting the Aruco markers. We obtain the grayscale image using the algorithm indicated in the configuration tool shown in Figure 2b. Figure 2d shows the resulting grayscale image binarization using the adaptive mean thresholding technique indicated in the configuration tool. We configure the block size in pixels (Binariz. Block Size) to apply the adaptive thresholding. We can detect numerous polygons in the walls of the swimming pool because they have mosaic tiles. We only extract the polygons with an area higher than the parameter configured on-line. We indicate this parameter as (Min. Polygon Area) in the configuration tool. Another filter consists of only considering quadrilateral polygons with edge length higher than the parameter (Min. Quad Side) configured on-line. Figure 2e shows the polygon filtering and rejection, where the yellow square indicates the area to remove the perspective projection computing the homography matrix. We depict the projected and binarized image in the upper left of the configuration tool of Figure 2b, which is tessellated into a regular grid to compare it with the dictionary of Aruco markers. We then perform the matching with the possible patterns in the four possible orientations. Once the landmark is detected, we calculate the area in pixels squared of the perceived Aruco marker because we use this magnitude to estimate the distance to the perception.

**Figure 3:**
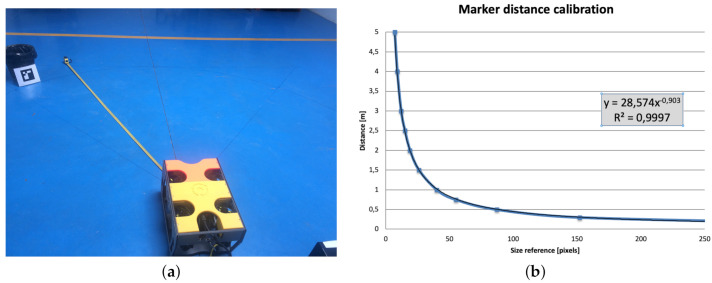
(**a**) Distance calibration of Aruco markers and (**b**) the distance calibration interpolation function.

The navigation system requires the distance estimation from the camera to the landmarks. The problem is not straightforward since the marker orientation can be any, as seen from the robot camera. Moreover, measuring the side of the detected polygon is not robust enough for distance triangulation. Lastly, but no less important, the camera lens distortion is quite appreciable, which is especially critical in non-centered perceptions. We solve these problems using a non-linear model to estimate the distance to the marker based on a measurable parameter of the detected Aruco model: the square root of the number of pixels contained in the polygon of the marker detection. A key issue is the calibration of the model. We proceed with a measurement process in which we measure this size reference to the marker positioned at different known distances. Finally, we produce an interpolation function that provides a distance estimation from the computed size reference. This process performs quite well if the marker is in the center of the image. Otherwise, the distance is undervalued. We calibrate the distance estimation using the following expression

(1) $$Distance\left(x\right)=a_{1}x^{b_{1}},\;with\left\{\begin{array}{c} a_{1}=28.2239\\ b_{1}=-0.903 \end{array}\right.$$

where *x* is the size reference obtained from the square root of the number of pixels contained in the polygon of the marker detection. We adjust $a_{1}$ and $b_{1}$ to obtain a coefficient of determination as close as possible to one ($R^{2}=1$) in the exponential fitting using least squares. Figure 3a shows the setup for the distance calibration process. Figure 3b shows the distance calibration using the non-linear interpolation function. We calibrate the uncertainty of distance estimation depending on the slope of the distance calibration interpolation function. We can observe a steeper slope with longer distance estimation.

**Figure 4:**
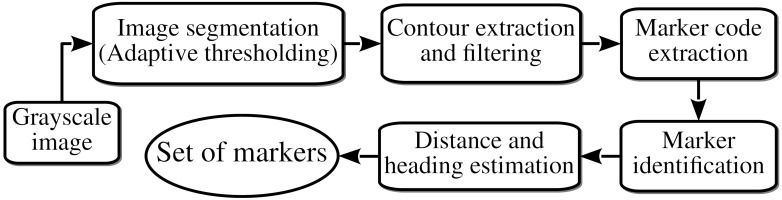
Flowchart of the procedure for detecting the Aruco markers surrounding the underwater vehicle.

Figure 4 summarizes the flowchart of the procedure for detecting the Aruco markers surrounding the underwater vehicle. The algorithm for detecting the landmarks operates with grayscale images. The first stage consists of the image binarization using an adaptive thresholding technique. We then extract the four-vertices polygons filtering the ones that do not satisfy some geometrical requirements. We detail the filtering criteria and the parameters for their configuration above. We remove the perspective projection of the candidate four-vertices polygons computing the homography matrix. We tessellate the resulting image using a regular grid to match such a resulting image and the reference Aruco markers in the possible orientations. Finally, we use the square area in pixels of the perceived landmark to estimate the distance, whereas we calculate the heading using the position of the detected marker in the image. This processing provides us the set of perceptions from each camera image.

### 3.2. Feature Extraction Using Sonar Scanner Readings

The information received from the mechanical scanning sonar is innate noisy because these active acoustic devices estimate the distance using the time-of-flight principle. Echoes from sonar are affected by different sources of uncertainty that can seriously degrade the distance estimation accuracy. Some examples are the wide opening angle of acoustic signals presented by most sonar sensors and the multi-path reflections. One solution to filter out noisy distance estimation readings is to check the coherence of data received at different times. We deal with this problem by building the spatio-temporal relations between the sonar echoes. Maintaining the sonar buffer implies a series of operations:

- **Aging**. We remove from the buffer those echoes that are older than a given amount of time. This filter is of paramount importance because the uncertainty of the local position of the sonar echoes grows unbounded with time.
- **Motion**. Whenever the vehicle moves, all the echoes stored in the buffer have to be translated and rotated correspondingly. This update is key to maintaining a coherent representation of the environment.
- **Blanking**. When a new scan is available, remove previous echoes that lie inside the scanning zone. The application of this filter is crucial for eliminating noise from the sonar buffer.

**Figure 5:**
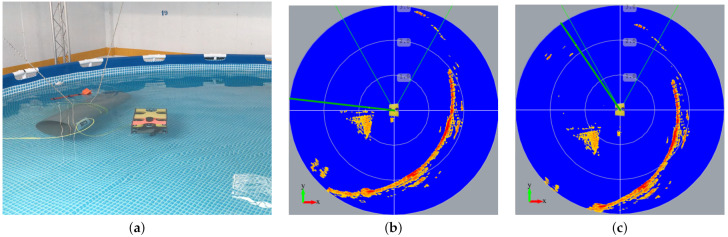
(**a**) Underwater vehicle with mechanical scanning sonar at the top, (**b**) local buffer of data received from the sensor, and (**c**) local buffer after the rotation and displacement of the underwater vehicle.

Figure 5a shows the underwater vehicle equipped with a mechanical scanning sonar at the top operating at a circular swimming pool. Figure 5b depicts the spatio-temporal buffer of sonar echoes. The distance estimation readings that lie along the green thick radial line are incorporated into the buffer while the underwater vehicle moves, performing both translations and rotations. The rotation velocity is computed from the heading readings, while we estimate the translation velocity from the inertial and DVL sensors. Echoes with the most amplitude are represented in red, while others in different shades of yellow. We can notice a static object close to the robot, about its left rear part, appears to move in the vehicle-centered coordinate space.

We can view the sonar buffer as a vehicle-centered consistent local map along time, at least up to a certain degree depending on how the vehicle’s velocity is measured or estimated. We can apply multiple feature extraction algorithms when a map is available. The next sections present the techniques adopted to perceive circumference arcs and line segments in structured environments. The perception of such features is useful for improving the accuracy of the navigation system.

#### 3.2.1. Circular Model-Fitting

The recognition of circumference arcs is useful when the underwater vehicle operates in a structured environment with these features. We can mention circular swimming pools, fish farms, and tanks, to name but a few. The recognition of these features provides us information that allows us to locate the vehicle to the distance estimated from the center of the circumference with a known location in the workplace. We can adopt different alternatives for the perception of circumference arcs [21] from the sonar scanner reading. We can mention algebraic-fitting methods and geometric-fitting techniques. The former is quite fast, but they lack robustness in the presence of outliers. The latter are iterative and tend to be robust in the presence of outliers. Since the sonar scanner readings tend to be quite noisy, algebraic fitting techniques produce very few fittings, always when the robot is static, and thus we conclude that geometric fitting methods seem more appropriate for circumference arc extraction. We follow the circle-fitting approach [22], which is detailed as follows.

Let  $P={\left\{\;\left(\;x_{i}\;,\;y_{i}\;\right)\;\right\}}_{i}$  be a set of points with a distribution approximately circular. We can use the following circle equation to model their position

(2) $${\left(\;x\;-\;a\;\right)}^{2}\;+\;{\left(\;y\;-\;b\;\right)}^{2}\;=\;R^{2}\;,$$

where  (a,b)  is the center of the circle and  *R*  its radius. Each point  $\left(\;x_{i}\;,\;y_{i}\;\right)$  in our set  P  will approximately satisfy this equation. We can rewrite this approximate equation factorizing in terms that contain the model parameters  {a,b,R} ,  and terms that contain the position of each point  $\left(\;x_{i}\;,\;y_{i}\;\right)$ as follows

(3) $$\begin{array}{cc} {\left(\;x_{i}\;-\;a\;\right)}^{2}\;+\;{\left(\;y_{i}\;-\;b\;\right)}^{2}\; & \approx \;R^{2}\;\Rightarrow \\ \Rightarrow \;x_{i}^{2}\;+\;a^{2}\;-\;2\;a\;x_{i}\;+\;y_{i}^{2}\;+\;b^{2}\;-\;2\;b\;y_{i}\;\; & \approx \;R^{2}\;\Rightarrow \\ \Rightarrow \;\left(\begin{array}{ccc} \;x_{i} & y_{i} & 1\; \end{array}\right)\;\left(\begin{array}{c} 2\;a\\ 2\;b\\ \;R^{2}\;-a^{2}\;-b^{2}\; \end{array}\right)\; & \approx \;x_{i}^{2}\;+\;y_{i}^{2}\;. \end{array}$$

We can then transform our model-fitting problem into a *linear least-squares* problem. Note that, since we have three unknown variables (*a*, *b*, and *R*), we need three (or more) $n_{e}$ equations to obtain a determined (or overdetermined) system of linear equations; at least three points to determine the parameters of our model. Then, building the matrices

(4) $$A\;=\;\left(\begin{array}{ccc} \;x_{1} & y_{1} & 1\;\\ \;x_{2} & y_{2} & 1\;\\ \;\vdots & \vdots & \vdots \;\\ \;x_{n_{e}} & y_{n_{e}} & 1\; \end{array}\right)\;,$$

(5) $$b\;=\;\left(\begin{array}{c} \;x_{1}^{2}\;+\;y_{1}^{2}\;\\ \;x_{2}^{2}\;+\;y_{2}^{2}\;\\ \;\vdots \;\\ \;x_{n_{e}}^{2}\;+\;y_{n_{e}}^{2}\; \end{array}\right)\;,$$

we can compute the matrix  X  that minimizes the distance  ${∥\;A\;X\;-\;b\;∥}^{2}$  through

(6) $$\begin{array}{c} X\;=\;{\left(\;A^{T}\;A\;\right)}^{-1}\;A^{T}\;b\;. \end{array}$$

Finally, we can obtain the model parameters using the following relations:

(7) $$a\;=\;\frac{X_{1}}{2}\;,\;b\;=\;\frac{X_{2}}{2}\;,\;R\;=\;\sqrt{\;X_{3}\;+\;a^{2}\;+\;b^{2}\;}\;,$$

where {$X_{1}$, $X_{2}$, $X_{3}$} is the solution of Equation (6).

However, we often obtain measurements that do not fit with a distribution approximately circular. We should detect and filter out these measures to achieve a robust detection of circumference arcs in the structured environment. The random sample consensus (RANSAC) method [23] and its variants are fundamental tools for outlier rejection. In particular, we rely on the RANSAC variant maximum likelihood estimation sample and consensus method MLESAC [24] to design the outlier rejection method in the circular model-fitting approach. For this purpose, we define the cost function for the model parameters  a,b, and *R*  as follows:

(8) $$\begin{array}{c} e_{i}\;=\;|\;R\;-\;\sqrt{{\left(x_{i}-a\right)}^{2}\;+\;{\left(y_{i}-b\right)}^{2}\;}\;|\;, \end{array}$$

(9) $$\begin{array}{c} \rho \left(\;e_{i}\;\right)\;=\;\left\{\begin{array}{cc} \;\;e_{i} & \mathrm{if}\;e_{i}<T\;,\\ \;\;T & \mathrm{if}\;e_{i}\geq T\;, \end{array}\right. \end{array}$$

(10) $$\begin{array}{c} C\left(\;a\;,\;b\;,\;R\;\right)\;=\;\sum_{i}\;\rho \left(\;e_{i}\;\right)\;. \end{array}$$

Algorithm 1 presents the circular model-fitting with outlier rejection. The algorithm requires the set of P points received from the mechanical scanning sonar (Blue Robotics Ping 360), the threshold *T* used to compute the cost function, the probability of not finding a correct model, and the proportion of inliers in data. The output of the process is the best model parameters for  a,b, and *R*.

**Figure 6:**
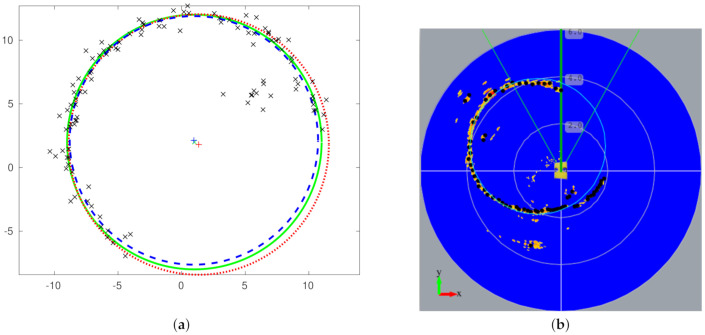
(**a**) Example of circle-fitting using noisy data, and (**b**) the fitted circumference using data received from the mechanical scanning sonar in a swimming pool with Algorithm 1.

Figure 6a shows an example of a fit to a circle with noisy data using Algorithm 1. We represent the set of points P to fit using black crosses. The continuous green circumference is the target fit with the center at the green cross point. We show the best circle-fitting using three points with a red dotted circle with the center at the red cross point. Finally, we depict the best fit using all the inliers with a dashed blue circumference with the center at the blue cross point. We can observe that the best fit using all the inliers is closer to the target solution than the fit using three points. Figure 6b shows the resulting circumference using the mechanical scanning sonar readings while the underwater vehicle navigates in the swimming pool.
**Algorithm 1** Circular model-fitting with outlier rejection**Input:**
 points▹ Set of points to be fitted T▹ Threshold used to compute the cost function FAILURE_PROBABILITY▹ Probability of not finding a correct model INLIER_PROPORTION ▹ Proportion of inliers in data**Output:**
 best2_a,best2_b,best2_R▹ Best model parameters found Initialization
1: $best\_C\;\leftarrow \;10^{12}$▹ Initialize to a large number2: $N\;\leftarrow \;\log\left(FAILURE\_PROBABILITY\right)/\log\left(1-INLIER\_PROPORTION^{3}\right)$ Find model
3: **for**i=1 to *N*
**do**
  Find possible model
4: Take 3 points randomly
5: Build matrices A and b using equations (4) and (5) and the 3 sampled points6: Find model parameters  a,b,R  using equations (6) and (7)7: Compute the cost function  *C*  using equations (8) to ()  If this possible model is better than the previous one, we keep it8: **if** (C<best_C) **then**
9:  best_C←C
10:  best_a,best_b,best_R←a,b,R11: **end if**
12: **end for**
 Refine the model using inliers13: Select the points  $\left(\;x_{i}\;,\;y_{i}\;\right)$  such that  $e_{i}<T$  using (8)14: Build matrices A and b using equations (4) and (5) and the selected inliers15: Find model parameters  best2_a,best2_b,best2_R  using equations (6) to (7)16: **return**
best2_a,best2_b,best2_R


#### 3.2.2. Line Segment Model-Fitting

The perception of line segments in structured environments is a more complicated task than the circle fitting presented above. This fact is because we can obtain several features of this type from the sonar scanning sensor. We have to deal with the uncertainty of such perceptions to maintain a coherent representation of the environment surrounding the underwater vehicle. We adopt a fuzzy segment framework [25,26] to represent and deal with the location uncertainty using line segments. These features include a representation of their uncertain location. The fuzzy segment framework represents the uncertainty using a fuzzy set whose degree of membership reflects how much the location could be occupied. This fuzzy segment framework provides power tools, based on similarity interpretation of fuzzy logic [27], to match the degree of similarity of information expressed as fuzzy segments. We use such tools to fuse and manage formally the uncertainty of the observations represented by fuzzy sets [28].

Let a line segment S be defined as a tuple as

(11) $$S=\{\theta ,\rho ,\left(x_{i},y_{i}\right),\left(x_{j},y_{j}\right),k\},$$

where θ and ρ are the parameters of the line equation xcos(θ)+ysin(θ)=ρ obtained by fitting *k* collinear range sensor observations, and $\left(x_{i},y_{i}\right)$ and $\left(x_{j},y_{j}\right)$ are the end-points of the line segment calculated as the projection of the sensor observations on the fitted line using the *k* collinear sensor observations.

We need to extract the set of *k* collinear sensor scanner readings to perform the eigenvector line fitting mentioned above. We have adopted an optimized algorithm that only split sets from consecutive reading. The main reason is the performance constraints of our application. In particular, we use the Iterative End Point Fit (IEPF) algorithm [29,30], which requires the initial definition of the minimum number of points $k_{min}$ of a set of collinear observations and the maximum distance $\rho_{max}$ of the scatter sensor readings to the fitted line segment. We have to remark that this algorithm requires a set of ordered observations. For a set *s* of continuous sensor scanner readings, the algorithm is as follows:

1. **Initialization**. We initialize the algorithm with a set *s* containing all the ordered observations.
2. **Step 1**. If the set *s* is composed of more than $k_{min}$ observations, draw a line segment between the first and last data (end-points), otherwise reject the set *s*.
3. **Step 2**. Detect the point *P* with maximum distance $\rho_{P}$ to the fitted line segment between the end-points.
4. **Step 3**. If $\rho_{P}\geq \rho_{max}$ splits the set *s* at *P* into two subsets $s_{1}$ and $s_{2}$ and **goes to Step 1** for both subsets. Otherwise, the set *s* is a candidate to be a line segment.
5. **Stopping criteria**. We finalize the search when all the subsets are a candidate to be a line segment satisfying the condition $\rho_{P}\leq \rho_{max}$ or are rejected because they have fewer than $k_{min}$ observations.

**Figure 7:**
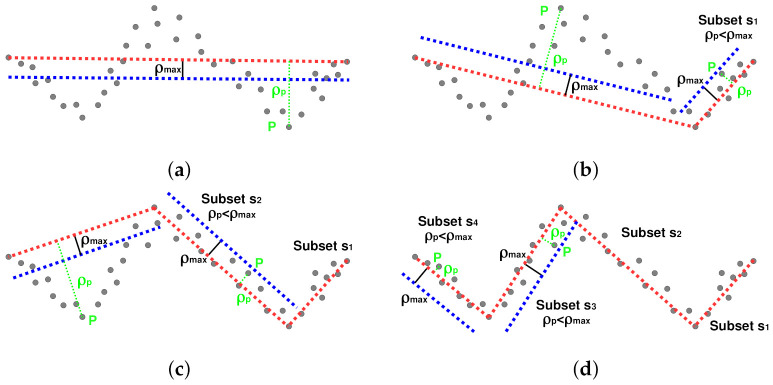
Iterative End Point Fit (IEPF) algorithm: (**a**) initial splitting process considering *k* points, (**b**,**c**) recursive split, and (**d**) stopping criterion.

Figure 7 shows an example of the Iterative End Point Fit recursive (IEPF) method. We can observe that the algorithm operates with a set *s* of *k* continuous sensor scanner readings. The initial step consists of drawing a line segment (represented as a red dotted line) between the end-points of the initial set *s*. Since there are numerous sensor readings further away from the confidence interval defined by the $\rho_{max}$ parameter (represented as a blue dotted line), the initial set is divided by the point *P* at the furthest distance $\rho_{P}$ from the line segment between end-points (red dotted line) into two subsets. If all the sensor readings of the corresponding set (with more than $k_{min}$ observations) are inside the confidence interval, we consider such a set of sensor readings as a candidate to be fitted as a line segment. We repeat this procedure until there is not any set candidate to form a line segment. We have to remark that this approach does not provide a set of line segments but groups of sensor readings candidate to be fitted as line-segments.

**Figure 8:**
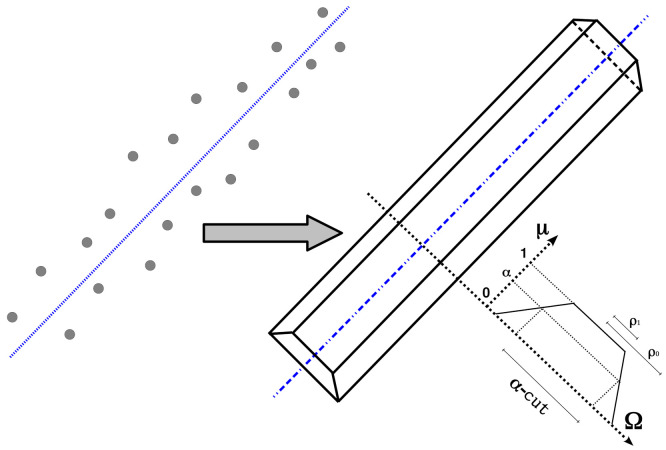
Scatter of points and fuzzy segment representing its uncertainty with a trapezoidal fuzzy set.

We represent the uncertainty on the location of the line segment *S* using the trapezoidal fuzzy set $t_{\rho }$. This set represents the uncertainty in the ρ parameter. Different factors can affect the location uncertainty of the ρ parameter, such as the line segment fitting from scattering sonar scanner readings, the aging of the fuzzy segment building, and the motion of the underwater vehicle, to name but a few. Assuming the independence of all these factors, we can define the trapezoidal fuzzy set $t_{\rho }$ in the Ω domain as the addition of the representation of all the sources of uncertainty that affect the ρ parameter as

(12) $$tp_{\rho }=tp_{\rho }^{1}⊕tp_{\rho }^{2}⊕...⊕tp_{\rho }^{n}=\left(-\rho_{0},-\rho_{1},\rho_{1},\rho_{0}\right),$$

where $tp_{\rho }^{i}$ with i=1,⋯,n are the trapezoidal fuzzy sets representing the *i* factors that influence the ρ parameter, ⊕ is the bounded sum operator, ($-\rho_{0}$,$\rho_{0}$) is the α-cut in the fuzzy membership μ=0, and ($-\rho_{1}$,$\rho_{1}$) is the α-cut in fuzzy membership μ=1. These α-cuts define the regions within fuzzy segments are considered within the degree of similarity α. We can use this criterion to address matching problems taking into account the location uncertainty. Figure 8 shows the scatter points fitted to a fuzzy segment with the trapezoidal fuzzy set $tp_{\rho }$, defined as an ordered tuple $\left(-\rho_{0},-\rho_{1},\rho_{1},\rho_{0}\right)$, including the different factors affecting the location uncertainty.

Thus, we define a fuzzy segment as a line segment *S* including its associated location uncertainty represented as a trapezoidal fuzzy set $t_{\rho }$ as

(13) $$FS=\{\theta ,\rho ,tp_{\rho },\left(x_{i},y_{i}\right),\left(x_{j},y_{j}\right),k\},$$

where $tp_{\rho }$ is the trapezoidal fuzzy set representing the uncertainty in ρ. We build this fuzzy set from the sonar scanner readings candidate to be fitted as a line segment. In particular, we assign the interval with confidence level 0.68 to the α-cut in μ=1, and the interval with a confidence level of 0.95 to the α-cut in μ=0. These intervals are the values of one and two standard deviations for a Gaussian distribution of the observations. The confidence interval for the Gaussian distribution with known variance is given by $\rho \pm |t_{k-1;1-\frac{\alpha }{2}}|\cdot \sigma_{\rho }$, where $t_{k-1;1-\frac{\alpha }{2}}$ is the value of a t-student distribution with k−1 degrees of freedom with probability $\frac{\alpha }{2}$ and $\sigma_{\rho }$ is the standard deviation of the fitted ρ parameter. Thus, the fuzzy set that represents the uncertainty of the fitted line is given by

(14) $$tp_{\rho }=\left(-\right|t_{0.025}|\cdot \sigma_{\rho },-|t_{0.16}|\cdot \sigma_{\rho },|t_{0.16}|\cdot \sigma_{\rho },|t_{0.025}\left|\cdot \sigma_{\rho }\right).$$

We can calculate the uncertainty in the θ parameter of the line equation xcos(θ)+ysin(θ)=ρ obtained by fitting *k* collinear range sensor observations as

(15) $$tp_{\theta }=\left(\theta -atan\left(\frac{2\rho_{0}}{l}\right),\theta -atan\left(\frac{2\rho_{1}}{l}\right),\theta +atan\left(\frac{2\rho_{1}}{l}\right),\theta +atan\left(\frac{2\rho_{0}}{l}\right)\right),$$

where *l* is line segment length.

We can maintain a coherent representation surrounding the underwater vehicle with the fuzzy segments using a similar approach to the Spatio-temporal relations between the different sonar echoes presented above. We can update the location and uncertainty of such features using the time elapsed from their generation. We can also update their position using the motion estimation of the underwater vehicle. The bounded sum operator ⊕ allows us to fuse the uncertainty of the *fuzzy segment* $tp_{\rho }$ with the motion estimation and the elapsed time from the generation of the features representing the world around the vehicle.

We can also fuse the detection of new features to the local perception representing the environment surrounding the underwater vehicle using the degree of similarity between fuzzy segments. We merge similar features by detecting their collinearity and fusing their uncertainty. Two segments $FS_{a}$ and $FS_{b}$ are considered collinear if they satisfy

(16) $$\begin{array}{ccc} & & f\left(tp_{\theta_{a}},tp_{\theta_{b}}\right)\geq 0.5\;\wedge \;f\left(tp_{\rho_{a}},tp_{\rho_{b}}\right)\geq 0.5, \end{array}$$

where f(x,y) function is the matching degree between two trapezoidal fuzzy sets defined in the same universe Ω as follows

(17) $$f\left(x,y\right)=\frac{\left(A_{x}+A_{y}\right)\cdot A_{xy}}{2\cdot A_{x}\cdot A_{y}},$$

where $A_{x}$ and $A_{y}$ denote the area enclosed by the fuzzy sets *x* and *y*, respectively, and $A_{xy}$ denotes the area of the intersection of *x* and *y*.

We combine new fuzzy segment perceptions with the ones contained in the buffer representing the environment around the underwater vehicle that satisfies the collinear condition (16). This procedure allows us to enrich the local representation and remove redundant information, which reduces the uncertainty of old and imprecise feature representations. The combined fuzzy segment $FS_{r}$ from two collinear ones is calculated by

(18) $$FS_{r}=\left\{\theta_{r},\rho_{r},tp_{\rho_{r}},\left(x_{ir},y_{ir}\right),\left(x_{jr},y_{jr}\right),k_{a}+k_{b}\right\},$$

where ($x_{ir}$,$y_{ir}$) and ($x_{jr}$,$y_{jr}$) are the end-point perpendicular projections of ($x_{ia}$,$y_{ia}$), ($x_{ja}$,$y_{ja}$), ($x_{ib}$,$y_{ib}$), and ($x_{jb}$,$y_{jb}$) on the line with ($\theta_{r}$,$\rho_{r}$) parameters calculated as

$$\begin{array}{ccc} \theta_{r} & = & \frac{k_{a}\theta_{a}+k_{b}\theta_{b}}{k_{a}+k_{b}},\\ \rho_{r} & = & \frac{k_{a}\rho_{a}+k_{b}\rho_{b}}{k_{a}+k_{b}},\\ tp_{\rho_{r}} & = & \left(2-f\left(tp_{\rho_{a}},tp_{\rho_{b}}\right)\right)\frac{k_{a}tp_{\rho_{a}}⊕k_{b}tp_{\rho_{b}}}{k_{a}+k_{b}}, \end{array}$$

where $tp_{\rho_{r}}$ is the trapezoidal fuzzy set representing the uncertainty of the fusedfuzzy segment.

**Figure 9:**
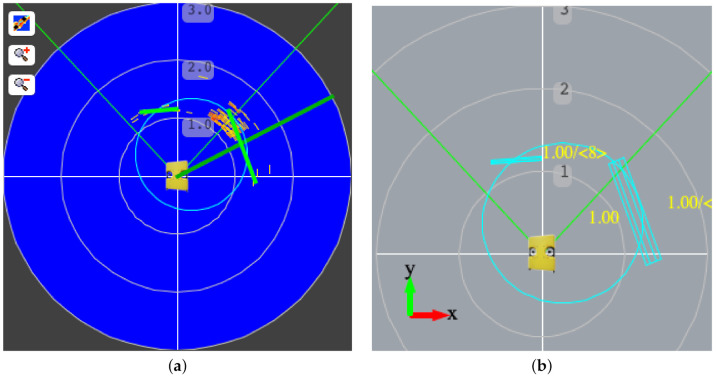
(**a**) Local buffer of sonar scanner data with line segment fitting and (**b**) fuzzy segment representation around the underwater vehicle.

Figure 9a shows an example of the buffer data using the range observations from the mechanical sonar scanner sensor. We extract the set of candidate points to be considered a line segment using the IEPF method. We fit these candidates to form a line using an eigenvector line fitting method. We represent these line segments using green lines. Figure 9b depicts the local representation of the environment around the vehicle using *fuzzy segments*. We can observe that such features include the location uncertainty using the corresponding trapezoidal fuzzy set $tp_{\rho }$. This local representation using fuzzy segments allows us to add and remove perceptions using a formal model, maintaining a coherent representation around the vehicle.

**Figure 10:**
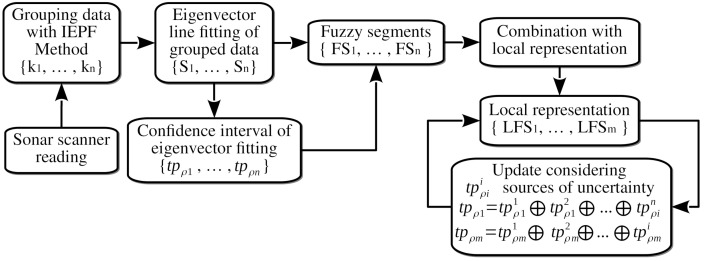
Flowchart of the procedure for building the fuzzy segment representation surrounding the underwater vehicle.

Figure 10 shows the flowchart of the procedure for building and maintaining a local representation of the environment using fuzzy segments. We group the sonar scanner readings into *n* sets with $\left\{k_{1},\cdots ,k_{n}\right\}$ observations using the IEPF method described above. We then fit such groups of consecutive sensor readings using some eigenvector line fitting method to obtain the set of line segments $\left\{S_{1},\cdots ,S_{n}\right\}$. We use the confidence interval of the line-fitting algorithm to build the trapezoidal fuzzy sets $\left\{tp_{\rho 1},\cdots ,tp_{\rho n}\right\}$ representing the uncertainty of the fuzzy segment with (14). Once we have calculated the set of *n* fuzzy segments detected from the observations, they are fused with the set of *m* fuzzy segments representing the environment around the underwater vehicle using the (16) criteria, or they are incorporated into such a representation. We update periodically the set of fuzzy segments $\left\{LFS_{1},\cdots ,LFS_{m}\right\}$ representing the local environment around the vehicle introducing the different sources of uncertainty affecting them. We model the uncertainty of the vehicle motion and the aging of the representation using trapezoidal fuzzy sets. We incorporate these sources of uncertainty into the fuzzy segment representation using the bounded sum operator of (12). We also remove these uncertain features when the area enclosed by the trapezoidal fuzzy set $tp_{\rho }$ of the fuzzy segment is higher than a prescribed threshold.

## 4. Particle Filter

**Figure 11:**
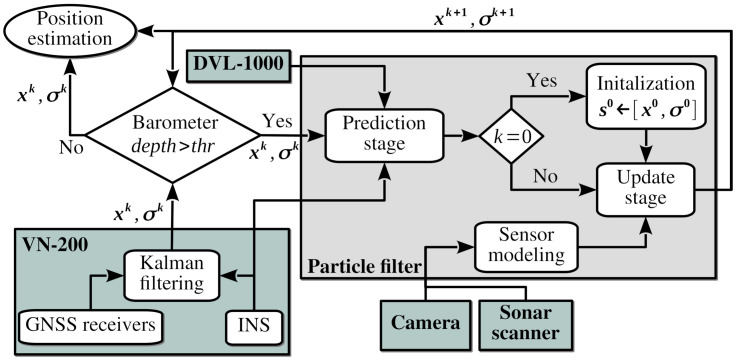
Flowchart of the navigation system.

We detail the flowchart of the navigation system in Figure 11. The localization system makes use of the GPS location provided by the Vectornav VN-200 navigation system. This device combines an inertial solid-state microelectromechanical system (MEMS) with a high-sensitivity GNSS receiver using Kalman filtering algorithms to estimate the position, velocity, and orientation. While the vehicle is on the surface, the navigation system makes use of the GPS. When the vehicle detects that it dives, by using the barometer of the standard Sibiu Pro platform, the last known and high-quality GPS position (using the HDOP Horizontal Dilution of Precision) is stored as a reference. The GNSS receiver still provides locations at low depths, but the position estimation degrades severely. Thus, we ignore GPS information when the barometer depth is higher than a threshold thr, for instance, thirty centimeters for the standard Sibiu Pro platform. We initialize the structured representation of the environment where the vehicle operates using a reference to the UTM (Universal Transverse Mercator) location where the vehicle submerged. From here on, the underwater localization method works in local metric coordinates. We convert these local estimations to global positions using the reference UTM position and the local coordinates. We can then convert the resulting UTM positions to latitude/longitude coordinates for visualization purposes. When the vehicle emerges again, we switch to GPS positions.

When the vehicle is operating submerged, we use a particle filter or Sequential Monte Carlo (SMC) method to fuse the proprioceptive and exteroceptive sensory information to estimate the location. SMC method estimates a variable of interest, typically with non-Gaussian and potentially multi-modal Probability Density Function (PDF) [31], in dynamical systems with partial observations and random perturbations, both in the measurements and in the dynamical system. The technique uses a set of particles (also called samples), with a likelihood weight representing the probability of that particle being sampled from the PDF, to represent the stochastic distributions of the state-space model and the noisy and partial observations. We can obtain an estimate of the variable of interest by the weighted sum of all the samples. The particle filter is recursive in nature operating in two phases: prediction and update. The former modifies the particles according to the acting model (prediction stage) and also incorporates random noise on the variable of interest. The latter re-evaluate the weight of samples $w_{i}$ using the sensory information available (update stage). We evaluate the particles periodically to remove particles with small weights. These samples have a low probability of being a sample from the PDF. This procedure is called *resampling*. Resampling techniques aim to avoid weight disparity, and thus the particles with negligible weight are replaced by new particles in the proximity of samples with higher weights.

Let $x^{k}={\left[p^{k},\theta^{k}\right]}^{T}$ be the state-space at the time *k* of the submerged underwater vehicle, where $p^{k}$ = ($x^{k}$, $y^{k}$) is the 2D location and $\theta^{k}$ is the vehicle’s orientation. We represent the 2D location $p^{k}$ uncertainty of each particle by a 2D Gaussian function, whose distribution follows a multivariate normal distribution $\phi \left(p^{k}\right)∼N_{2}\left(p^{k},\sigma \right)$ with σ the correlation coefficient between ($x^{k}$, $y^{k}$) variables. This representation allows us to model the location uncertainty of the perceptions and then merges it with the set of particles representing the probability density function of the variable of interest [32]. It also allows us to evaluate the probability of the particles representing the PDF, which is used to reject them at the resampling stage.

Thus, we represent the location of the underwater vehicle (variable of interest) as a set of *n* particles $s_{i}^{k}=\left[x_{i}^{k};w_{i}^{k};\phi_{i}^{k}:i=1,\cdots ,n\right]$, where: the index *i* denotes the sample (copies of the variable of interest), the weight $w_{i}$ defines the contribution of the particle *i* to the overall estimate of the variable of interest, and the density function $\phi_{i}$ represents the 2D location uncertainty ($x^{k}$, $y^{k}$) of each particle *i* to the estimation of the location uncertainty. Algorithm 2 presents a pseudo-code of the recursive estimation process of the state-space using the particle filter. When the vehicle submerges, we set the time step *k* to zero and initialize the set $s^{k}$ of *n* particles considering the last location *p* and uncertainty provided by the Vectornav VN-200 navigation system using the GNSS receiver and the inertial navigation system (INS). In particular, we initialize the location uncertainty with correlation coefficient σ to all the samples, which are randomly distributed around the position estimation *p* of GPS depending on the accuracy of such an estimation.

Then, the localization algorithm estimates the variable of interest $x^{k}$ recursively by the *prediction* and *update stages*. The former incorporates the motion estimation α, provided by the INS and DVL devices, to all the particles representing the location belief. We also include a certain degree of random noise configured by a normal distribution with variance $\alpha_{u}$ to spread the particles. The spreading of particles contributes to a better representation of the vehicle belief since the resampling duplicates samples with high weight. The latter updates the weights $w^{k}$ of the set of particles $s_{i}^{k}$ representing the vehicle belief by the product of the 2D Gaussian distribution of the features detected γ and the $\phi^{k}$ distribution of each sample. We obtain such a weight from the resulting likelihood of the product operation between two 2D Gaussians representing the location uncertainty. This approach allows us to merge the uncertainty of both sources: the sample and the perceived feature. The result of the product operation between two Gaussian distributions has a low likelihood for distributions representing different locations. Since these samples with low weight have a low probability of being a sample from the vehicle belief, we remove them from duplicating samples with high weight. These redundant particles have a different location when we apply the random noise of the prediction stage. There are different criteria to perform the resampling [33], we adopt the effective number of particles (ENP) as defined in Algorithm 2. When this number is lower than the product β·n, with β tuned for the particular application and *n* the number of samples, we perform the resampling of the set of particles. We depict the flowchart of all these steps in Figure 11. Finally, the particle filter approach allows us to estimate a position $x^{k}$ and its location uncertainty $\sigma^{k}$ from the vehicle belief by the weighted average of the samples and the correlation coefficient, respectively.
**Algorithm 2:** Particle filtering for localization. Initialization
1: $p_{i}^{0}\leftarrow N_{2}\left(p,\sigma \right)\;\left\{i=1,\cdots ,n\right\}$▹ Randomly initialization of particles from location *p*2: $\phi_{i}^{0}\;\leftarrow N_{2}\left(p_{i}^{0},\sigma \right)\;\left\{i=1,\cdots ,n\right\}$▹ Initialization of distribution from the position $p_{i}^{0}$3: $s_{i}^{0}\;\leftarrow \;\left[x_{i}^{0};w_{i}^{0};\phi_{i}^{0}:i=1,\cdots ,n\right]$▹ Initialization of samples within the uncertain location Recursive loop for localization
4: **while** true **do**
5: k++
6: ENP = $\frac{1}{\sum_{i=1}^{n}log^{2}\left(w_{i}^{k}\right)}$▹ Effective number of particles7: **if** ENP < β·n**then**▹ Condition of particle population depletion (0≤β≤1)8:  $s^{k}$← Resampling($w^{k}$)9: **end if**
10: *Prediction stage*
11: $x^{k+1}$←$h(x^{k},\alpha )$▹ Include action α (dead-reckoning displacements)12: $x^{k+1}$←$x^{k+1}$+α·$N(0,\alpha_{u})$▹ Include ramdom noise to the variable of interest13: *Update stage*
14: $w^{k+1}$=$w^{k}\cdot g\left(\gamma ,x_{j}^{k}\right)$▹ Update with sensing γ15: Normalization of the weights
16: **for**
*j*← 1 to *n*
**do**
17:  $w_{j}^{k+1}$=$\frac{w_{j}^{k+1}}{\sum_{i=1}^{n}w_{i}^{k+1}}$18: **end for**
19: **end while**


## 5. Experimental Results

We have conducted experiments in two different scenarios to evaluate the performance and accuracy of the proposed methods. One set of experiments have been carried on in a controlled environment, a circular swimming pool, while the other set of experiments is carried on at sea, in a harbor dock. The former scenario allows us to evaluate the sensor modeling and localization in a structured environment. We have an external vision system that tracks the position of the vehicle in the controlled environment. This location estimation serves as a ground-truth and allows us to correlate the position estimates using the navigation system and the ground-truth. The latter scenario presents the navigation system operating in a more complex scenario without any system to estimate the ground-truth. In this case, we evaluate the accuracy of the system comparing the last estimated underwater position with the first stable GPS position obtained when surfacing. Care has been taken to emerge vertically so that the error associated with emerging is negligible.

We perform the experiments running the intensive computation in a remote computer. We communicate with the Raspberry Pi using TCP/UDP protocols through the Ethernet of the umbilical cable. We also use this computer for monitoring, configuring, and operating the underwater vehicle. This computer installs an Intel Core i7 running at 3.3 GHz. We configure the image acquisition with 480 × 360 resolution, which allows us to compute 15 frames per second in the remote computer. In particular, vision processing takes about 25 milliseconds on average. Concerning the localization approach, it takes about three milliseconds per update. This timing is tessellating the variable of interest using 1000 samples. By adjusting this timing allows us to perform the localization update every 150 milliseconds.

### 5.1. Experiments in the Swimming Pool Scenario

**Figure 12:**
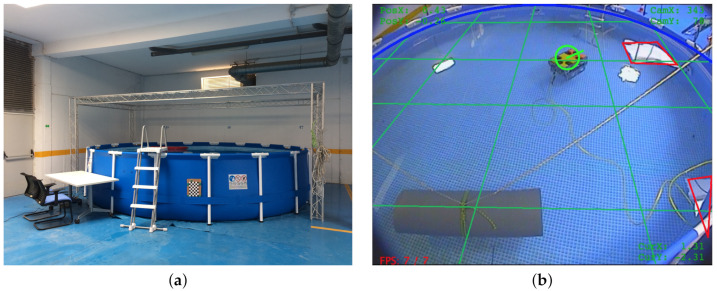
(**a**) Structured swimming pool scenario and (**b**) ground-truth estimation system.

The experiments in the swimming pool consist of the submerged navigation of the underwater vehicle performing inspection tasks. Figure 12 shows the swimming pool scenario and the external vision system designed to provide the ground-truth. The swimming pool has six meters in diameter and has different objects simulating working conditions for inspection tasks. The shallow depth of the swimming pool is enough to degrade the GNSS signal. Thus, the particle filter becomes mandatory for underwater navigation. The vehicle uses the mechanical sonar scanner Ping360 to perceive arcs corresponding to the swimming pool walls. We use the standard camera of the Sibiu Pro platform to detect the Aruco markers. We use the sensor modeling techniques presented above to estimate the features surrounding the vehicle during underwater navigation. We fuse these features with the motion estimation to calculate the vehicle belief using the particle filter.

**Figure 13:**
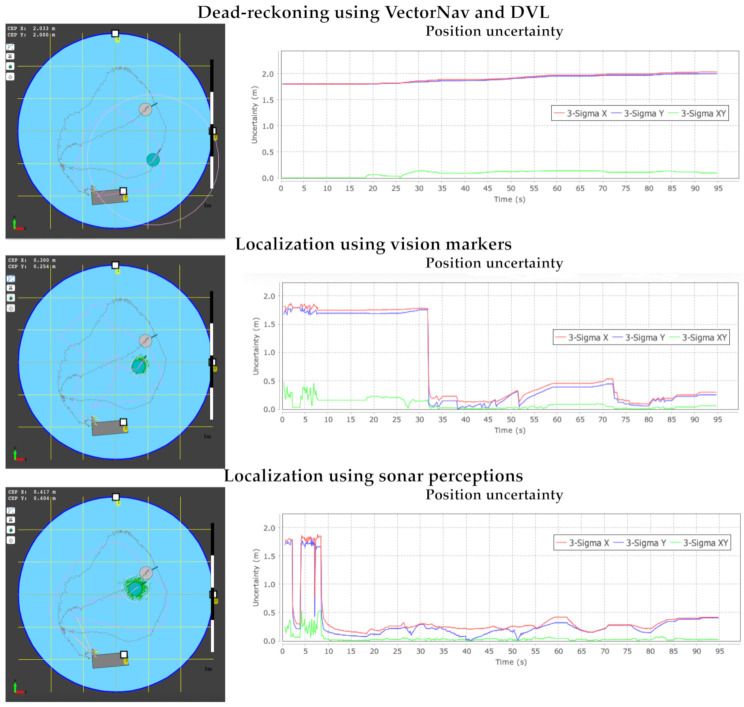
Position estimation uncertainty using (**top**) dead-reckoning, (**middle**) vision markers, and (**bottom**) sonar perceptions in the structured swimming pool scenario.

Figure 13 shows the path followed by the vehicle in underwater navigation performing inspection tasks. The brown circle represents the ground-truth position estimation using the external vision system, and the brown segment-lines the connection between location estimations. The green circle represents the location estimation using the corresponding sensory information. The pink line segments represent the connection between position estimates with the navigation system of the vehicle. We evaluate the estimated path followed by the immersed vehicle in three situations: navigation using dead-reckoning, using the vision markers, and using the sonar scanner observations. We can observe that the ground-truth is similar for the three cases since we represent the same experiment using different sensory information.

The dead-reckoning experiment only uses the INS of the Vectornav VN-200 navigation system and the DVL-1000 speed estimations. We can observe that the path followed using dead-reckoning approximates the route followed by the underwater vehicle. However, the position error is cumulative or compounding over time using such an approach. Thus, we can observe that the underwater vehicle goes through the pipe in the structured environment because the estimated position degrades over time. We also have noted that the correlation coefficient σ grows unbounded during the navigation. We only introduce three Aruco markers in the structured environment, which are detected sporadically during the navigation. We represent these Aruco markers using empty squares with the corresponding ID. These sporadic observations allow us to correct the position estimation and the uncertainty of the vehicle belief represented by the correlation coefficient σ, as shown in Figure 13(middle). However, we can observe that the position estimation compared to the ground-truth is of the order of half a meter. We have to remark that we can enhance the accuracy of position estimation by adding vision markers. The Video S1 in the Supplementary Materials shows the navigation and position estimates using the Aruco markers. Finally, we can observe that the best position estimation is obtained using the features from the sonar scanner observations, in particular the circumference arcs obtained using the circular model fitting presented above. The representation surrounding the vehicle during the underwater navigation allows us to feed the localization approach with coherent information that allows us to track the vehicle with a high degree of accuracy. We can also observe that the estimation σ of the uncertainty of vehicle location is kept under half a meter during the navigation. This information is coherent with the location estimation provided by the ground-truth.

### 5.2. Experiments in the Dock Harbor Scenario

**Figure 14:**
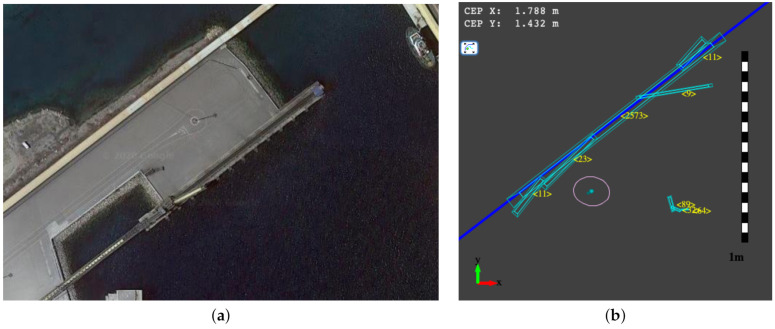
(**a**) Harbor dock scenario and (**b**) the fuzzy segment representation of the environment surrounding the vehicle.

The experiments in the harbor dock consist of the submerged navigation of the underwater vehicle performing inspection tasks. In particular, we follow the dock harbor wall to perform such inspection tasks [34]. Figure 14a shows the satellite image of the dock harbor scenario. Figure 14b depicts a representation of the environment around the vehicle using the *fuzzy segment* representation. We update the belief of the variable of interest using the speed estimates provided by the INS of the Vectornav VN-200 navigation system and the DVL-1000. The vehicle uses the mechanical sonar scanner Ping360 to perceive the walls of the dock harbor. We use the sensor modeling techniques presented above to perceive the features surrounding the vehicle during underwater navigation. We fuse these features with the motion estimation to estimate the vehicle belief and the location uncertainty using the particle filter.

**Figure 15:**
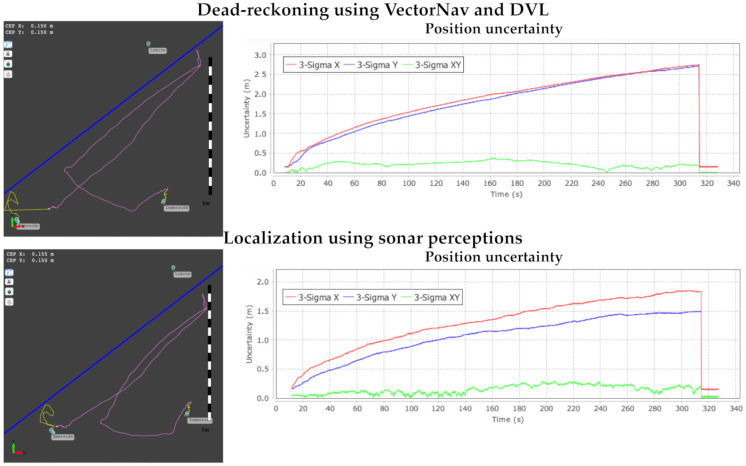
Position estimation uncertainty using (**top**) dead-reckoning and (**bottom**) particle-based localization system sensing uncertain line segments in the harbor scenario.

In this scenario, we do not have an external system providing the ground-truth. We only can evaluate the accuracy of the localization when the vehicle emerges. We do it by comparing the GPS location with the position estimation in submerged navigation. As previously mentioned, we initialize the structured representation of the environment from the last GPS estimation using UTM coordinates, and when the vehicle emerges, we transform the estimated underwater position in the structured local representation to UTM coordinates again. Figure 15 shows the position estimation of the submerged vehicle during underwater navigation. The yellow line segments represent the connection of position estimates using the GPS information, whereas the pink line segments represent the connection between position estimates in submerged conditions. The localization system performs this switch using the barometer readings, as shown in Figure 11.

Figure 15(top) shows the path followed using dead-reckoning with the speed estimation provided by the INS of the VectorNav and DVL-1000. We can observe that the correlation coefficient σ representing the location uncertainty grows unbounded during the navigation. We also note that the position estimation is drifting further and further away from the wall that it is inspecting. When the vehicle emerges, we observe that the GPS position is at a distance of more than five meters from the location estimated by the underwater navigation system. The drastic changes of the GPS position estimates are attributed to the initialization of the Kalman filters of the Vectornav VN-200 navigation system. Figure 15(bottom) shows the path followed by the underwater vehicle using the sonar scanner observations with the line fitting approach and the fuzzy segment representation. The representation around the underwater vehicle allows us to feed the localization approach with coherent information, which allows us to track the vehicle with a high degree of accuracy. We have to remark that a wall does not provide information to locate the vehicle, but ensuring that the vehicle location is posed at the corresponding distance from the wall. For these reasons, the estimation σ of the uncertainty of vehicle position is only reduced in one dimension. The localization algorithm would need more information to perform some kind of triangulation to locate the vehicle. In any case, the position estimated by the particle filter is located at a distance of less than one meter from the GPS location when the underwater vehicle emerges.

## 6. Conclusions and Future Works

We have presented the processing, modeling, and fusing of different underwater sensor signals to provide a reliable representation for underwater localization in structured environments. In particular, we have presented the feature extraction from the buffered data of underwater observations using a camera and a mechanical sonar scanner. The underwater sensor readings using these sensors are noisy and uncertain, and thus we propose a mechanism to verify such measures by coherent consecutive and redundant data observations, which are removed from the buffer by aging and disparity. We propagate the uncertainty of such perceptions to the set of features surrounding the underwater vehicle, which provides a coherent representation of the environment. We also update and remove these features using the factors previously mentioned. This processing filters out inconsistent information that can deteriorate the position estimations of the localization approach. We use the extracted features from noisy underwater sensors to feed the update stage of a particle filter localization method. However, we can also use the proposed sensor modeling techniques with other localization approaches. We evaluate the underwater sensor modeling with the accuracy of the localization system when the vehicle submerges. The experimental results show significant accuracy improvements in comparison with dead-reckoning underwater navigation. As future works, we plan to include acoustic sensor readings in the proposed framework, fusing these measurements with the perceptions using sonar and optical sensors. We also plan to estimate the location by combining a local (tracking) and a global localization method. This will allows us to improve the accuracy and robustness of the position estimation.

## Data Availability

We include a video showing the development working in real-world conditions.

## Supplementary Materials

The following are available at https://www.mdpi.com/1424-8220/21/4/1549/s1, Video S1.
